# Breast cancer molecular subtypes and receptor status among women at Potchefstroom Hospital: a cross-sectional study

**DOI:** 10.11604/pamj.2021.38.85.23039

**Published:** 2021-01-26

**Authors:** Baudouin Kongolo Kakudji, Prince Kasongo Mwila, Johanita Riétte Burger, Jesslee Melinda du Plessis, Kanishka Naidu

**Affiliations:** 1 Department of Surgery, Potchefstroom Hospital, Potchefstroom, North West Province, South Africa,; 2 Department of Surgery, School of Clinical Medicine, Faculty of Health Sciences, University of the Witwatersrand, Johannesburg, South Africa,; 3 Medicine Usage in South Africa (MUSA), Faculty of Health Sciences, North-West University, Potchefstroom, South Africa

**Keywords:** Breast cancer, molecular subtypes, receptor status, Potchefstroom, South Africa, cross-sectional study

## Abstract

**Introduction:**

this study aimed to determine the prevalence of receptor status and molecular subtypes in women with breast cancer treated at Potchefstroom Regional Hospital, South Africa and to analyze the association of molecular subtypes with some clinicopathologic characteristics of the tumor.

**Methods:**

the study population for this cross-sectional study consisted of 116 women with primary invasive breast cancer, treated at the hospital from 1^st^ January 2012 to 31^st^ December 2018. Molecular subtypes were classified by immunohistochemical surrogates as luminal A (estrogen receptor (ER) positive and/or progesterone receptor (PR) positive, HER2-; Ki-67 <30%), luminal B HER2- (ER+ and/or PR+, HER2-; Ki-67 ≥30%), luminal B HER2+ (ER+ and/or PR+, HER2+; any Ki-67), HER2 enriched (ER- and PR-, HER2+; any Ki-67), or triple-negative (ER-, PR-, HER2-, any Ki-67).

**Results:**

the proportions of breast cancer receptor status of ER+, PR+ and HER2-, were 71.6%, 64.7% and 75.9%, respectively. The molecular subtypes of 29.3% of patients were luminal A-type, 24.1% were luminal B HER2-, 22.4% were triple-negative, 18.1% were luminal B HER2+ and 6% were HER2-enriched. Molecular subtypes were significantly associated with tumor grade (p <0.001; Cramér's V=0.337), but independent of age (p=0.847), menopausal status (p=0.690), histology type (p=0.316), cancer stage (p=0.819), lymph node status (p=0.362), or tumor size (p=0.255).

**Conclusion:**

the study has revealed that most of the breast cancer in our setting was receptor-positive; approximately one-quarter were triple-negative. Furthermore, the study showed that luminal type A and B are the preponderant molecular subtypes. Molecular subtypes were associated with tumor grade but independent of age and menopausal status. The current study may assist in guiding the therapeutic strategy for patients with breast cancer in the Potchefstroom hospital catchment area.

## Introduction

The improvement in the knowledge of breast cancer over the last two decades has highlighted the importance of molecular subtypes in the understanding and management of breast cancer. It is common knowledge that breast cancer is a very heterogeneous disease, with heterogeneity between different subtypes and within the same molecular subtype [1]. Molecular subtypes influence the choice of therapy, determine the progression of the disease and predict the treatment response and long-term survival [2]. In this era of personalized cancer treatment, precise stratification of molecular subtypes permits patients with tumors of low proliferation rate and high expression of hormonal receptors to forgo adjuvant chemotherapy as it has little benefit on long-term survival and recurrence in this particular group [3].

Breast cancer subtypes can be identified and classified using immunohistochemistry (IHC) or more accurately, through micro-array-based gene expression profiling (GEP) [4]. In early 2000, progress was made in understanding the molecular heterogeneity of breast cancer on account of the seminal work of Perou *et al*. [5]. Using the GEP, they identified and classified four intrinsic molecular subtypes: luminal A, luminal B, human epidermal growth factor receptor 2 (HER2)-enriched and basal-like [5]. With the development of multi-gene expression studies, new concepts such as integrative clusters subtypes and theranostic therapy have emerged [6,7]. From the numerous multi-gene classifiers at disposal, there are the first-generation multigene tests commercially available that are used in clinical practice and the second-generation which is less expensive and provides better prediction of risk of recurrence, distant metastasis and response to chemotherapy in early breast cancer. These genomic signatures are influencing therapeutic choices and determining the risk of local recurrence in early breast cancer [8,9].

Due to a lack of resources, Africa is lagging in the integration of genomic markers in the management of breast cancer. Breast cancer is still treated based on the clinical, pathological and immunohistochemical characteristics of tumors [10]. Although GEP has been available in South Africa since 2007 [11], ER/PR mRNA reporting only became accessible in 2011 [12]. GEP is not readily available in South African public hospitals. To compensate for the lack of GEP, IHC combining estrogen receptor (ER), progesterone receptor (PR), HER2 and Ki-67 (a human nuclear antigen proliferative marker) is the best substitute [13].

Molecular subtype and receptor status studies are not extensively done in developing countries [14]. Therefore, this study aimed to depict the prevalence of receptor status and molecular subtypes in women with breast cancer treated at Potchefstroom Regional Hospital, South Africa and to analyze the association of molecular subtypes with other prognostic factors such as the age of the patients, menopause status with age as a proxy, stage of the disease, lymph nodes status, histological type, tumor grade, tumor size and Ki-67 proliferation index.

## Methods

**Figure 1:**
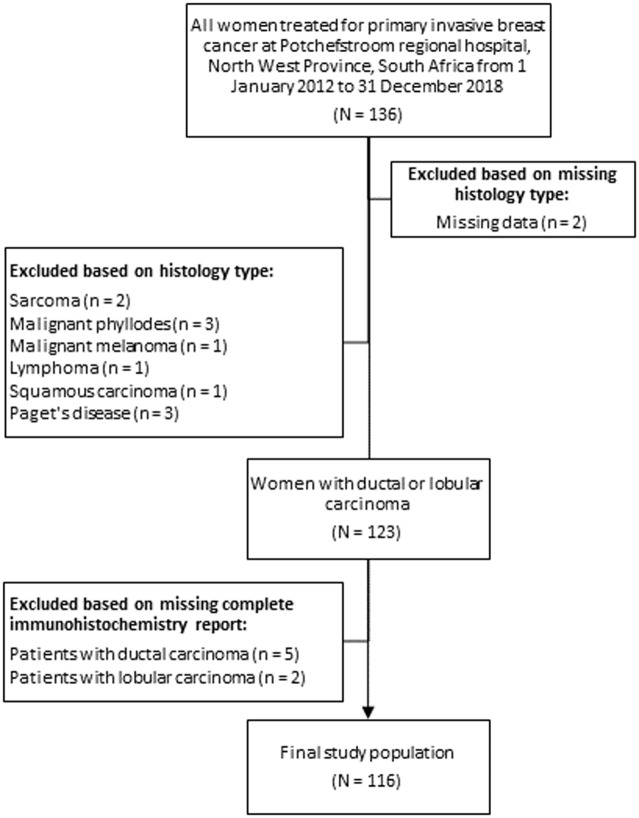
flow diagram illustration of the selection of participants

**Study design and study population:** this cross-sectional study describes retrospective data for patients with primary invasive (ductal or lobular carcinoma) breast cancer, receiving treatment at Potchefstroom Regional Hospital, North West Province, South Africa, from 1^st^ January 2012 to 31^st^ December 2018. From an initial total of 136 women who presented with primary invasive breast cancer at the hospital during the study period, two patients were excluded because of missing histology reports. Of the remaining 134 patients, 123 had ductal or lobular carcinoma. Only 116 of these patients had a complete IHC report available and were subsequently included in molecular subtype classification (Figure 1).

**Data source and data collection:** data were collected by triangulation from hospital registers (breast clinic, wards and theatre), patient files and histology reports. The IHC was part of routine histological reports obtained from the National Health Laboratory Service (NHLS) which adheres to a standard protocol for collecting, processing and reporting of specimens. Data were captured using a Microsoft Word® data collection tool. It took place from February 2019 to October 2019. The tool was converted to a Microsoft Excel® data capturing sheet. A 5% data re-entry method was followed, whereby 5% of the data were entered into a second dataset. The datasets were then compared electronically using the 'compare datasets command' in the Statistical Package for the Social Sciences (IBM SPSS® 25). Discrepancies flagged as errors were resolved manually by comparing the electronic dataset to the data collection tool, using the patient number indicated on the tool/sheet. The process was repeated until no discrepancies were found. Data were also checked for any outliers.

Data fields included in the study for each patient consisted of molecular subtype, patient age, breast cancer histological type, tumor size, disease stage, axillary lymph node involvement, tumor grade, receptor status (ER, PR and HER2) and Ki-67 proliferation index. Receptor status for tumors was abstracted from the standard histopathology results. ER, PR and HER2 were either positive or negative. As standard practice for the HER2, in case a result was inconclusive, the laboratory automatically performed an *in situ* hybridization test which had to confirm if the test was positive or negative. Molecular subtypes of breast cancer were based on the 2015 St Gallen International Expert Consensus´ definition [15], as indicated in Table 1. Patients were further categorized as either pre- or post-menopausal. Because of a lack of data in patient files on the actual age of onset of menopause, we used patients´ age as a proxy measure and considered all patients of 50 years and above as having natural menopause. This was done because most women in South Africa have their menopause between the ages of 45 and 50 years [16].

**Table 1:** molecular subtypes breast cancer based on 2015 St Gallen International expert consensus definition

Subtypes of breast cancer	ER and PR	HER2	Ki-67
Luminal A	ER+ and/or PR+	HER2-	Ki-67 <30%
Luminal B- Her2 NEG	ER+ and/or PR+	HER2-	Ki-67 ≥30%
Luminal B- Her2 POS	ER+ and/or PR	HER2+	Any Ki-67
Her2 enriched	ER- and PR-	HER2+	Any Ki-67
Triple-negative	ER- and PR-	HER2-	Any Ki-67

**Data analysis:** the objective of data analysis was to calculate the prevalence of breast cancer receptors status (ER, PR, HER2) and to analyze the association between molecular subtypes (luminal A, luminal B, HER2+, luminal B HER2-, HER2-enriched, triple-negative) and age, menopausal status, stage of the disease, lymph nodes status, histological type, tumor grade, tumor size and Ki-67 index. Data were analyzed using SPSS (IBM SPSS® 25). Tests for normality (Q-Q plots) were used to determine data distribution. Continuous variables were expressed as means, standard deviations and 95% confidence interval (CI) if normally distributed or medians and interquartile ranges if skewed. Categorical variables were expressed as counts and percentages. The analysis of variance (ANOVA) was used to determine the difference in the mean age of patients between molecular subtypes, whereas the difference in the mean Ki-67 index between molecular subtypes, stratified by menopausal status was determined using the student´s t-test. To determine the practical significance of differences between means, Cohen´s d was used. Cohen´s d was interpreted as follows: 0.2 was deemed as a small effect size, 0.5 as a medium effect size and 0.8 practically significant [17].

Pearson´s Chi-square/Fisher´s exact test was used to test for associations between molecular subtypes with menopausal status, histological type, cancer stage, axillary lymph nodes, tumor size and tumor grade. The one-sample Chi-square test was performed to test if single categorical variables followed a hypothesized population. A two-tailed p-value, where p<0.05 was considered significant, was used. Practical significance of associations was computed when p-values were significant. Cramér´s V statistic was used to determine the effect size of associations, with Cramér´s V equal to 0.1 deemed as a weak association, V value of 0.3 as a moderate association and V value of 0.5 regarded as a large effect/association.

**Ethical considerations:** permissions were obtained from the North West Provincial Department of Health and the Health Research Ethics Committee of the North-West University (NWU-00007-19-S1) to perform the study. Goodwill permission to conduct the study was sought from the Potchefstroom Hospital patient´s safety group.

## Results

The presenting characteristics of the study population are summarized in Table 2. A total of 116 women (mean age 56.35 (14.09) (95% CI, 53.76, 58.94)) met the inclusion criteria. Invasive ductal carcinoma (96.6%) was the most prevalent histology type. Using age as a proxy for menopausal status, the majority of patients in the study population (62.9%) were post-menopausal. The results showed that 71.6% of patients were ER+, 64.7% were PR+ and 75.9% were HER2 negative. The median (IQR) Ki-67 was 30.0% (15.0-42.0). Based on the Ki-67 cut-off of ≥30%, more than half of patients (55.2%) had a high proliferation index. We found 29.3% of patients had luminal A breast cancer, followed by 24.1% of patients with luminal B HER2-negative breast cancer, 22.4% with triple-negative breast cancer and 18.1% with luminal B HER2-positive breast cancer. Six percent of patients presented with HER2-positive enriched breast cancer (Table 2).

**Table 2:** demographic, clinical and immunohistochemical characteristics of the study population

	n	%
**Menopausal status**		
Pre-menopausal	43	37.1
Post-menopausal	73	62.9
**Histological type**		
Invasive ductal carcinoma (IDC)	112	96.6
Invasive Lobular carcinoma (ILC)	4	3.4
**Estrogen receptor status**		
Negative	33	28.4
Positive	83	71.6
**Progesterone receptor status**		
Negative	41	35.3
Positive	75	64.7
**HER 2 status**		
Negative	88	75.9
Positive	28	24.1
**Proliferation index**		
<30%	52	44.8
≥30%	64	55.2
**Molecular subtypes**		
Luminal A	34	29.3
Luminal B, HER2-positive	21	18.1
Luminal B, HER2-negative	28	24.1
HER2-enriched	7	6.0
Triple-negative	26	22.4

Table 3 displays patient and clinicopathologic parameter characteristics by molecular subtypes. Although molecular subtypes were independent of menopausal status (measured using age as a proxy measure) (p=0.690), patients with luminal molecular subtypes breast cancer (luminal A and luminal B) were marginally older than those with non-luminal breast cancer (triple-negative/HER2-enriched). There was no association between molecular subtypes and histology type (p=0.316), cancer stage (p=0.819), lymph node status (p=0.362), or tumor size (p=0.255). Patients had, irrespective of molecular subtypes, preponderance for positive axillary lymph nodes (67.0%).

**Table 3:** relation between clinicopathologic parameters and molecular subtypes

Molecular subtypes	Luminal A (N=34)		Luminal B, HER2+ (N=21)		Luminal B, HER2- (N=28)		HER2-enriched (N=7)		Triple-negative (N=26)		P-value
**Clinicopathologic parameters**	**n**	**%**	**n**	**%**	**n**	**%**	**n**	**%**	**n**	**%**	
**Menopausal status**											0.690*
Pre-menopausal	12	35.3	7	33.3	11	39.3	3	42.9	10	38.5	
Post-menopausal	22	64.7	14	66.7	17	60.7	4	57.1	16	61.5	
**Histological type**											0.316*
Invasive ductal carcinoma	32	94.1	21	100	26	92.9	7	100	26	100	
Lobular	2	5.9	0	-	2	7.1	0	-	0	-	
**Cancer stage**											0.819*
Stage I	3	8.8	2	9.5	2	7.1	1	14.3	3	11.5	
Stage II	8	23.5	5	23.8	11	39.3	1	14.3	8	30.8	
Stage III	18	52.9	7	33.3	5	17.9	3	42.9	9	34.6	
Stage IV	5	14.7	7	33.3	10	35.7	2	28.6	6	23.1	
**Axillary lymph nodes**											0.362*
Negative lymph nodes	11	32.4	4	19.0	12	42.9	1	14.3	9	34.6	
Positive lymph nodes	22	64.7	16	76.2	15	53.6	6	85.7	16	61.5	
Missing data	1	2.9	1	4.8	1	3.6	0	-	1	3.8	
**Tumor size**											0.255*
>2cm	4	11.8	1	4.8	2	7.1	1	14.3	5	19.2	
> 2 ≤ 5 cm	19	55.9	9	42.9	16	57.1	4	57.1	14	53.8	
> 5 cm	11	32.4	11	52.4	10	35.7	2	28.6	7	26.9	
**Tumor grade**											0.000*
Grade 1	6	17.6	0	-	0	-	0	-	1	3.8	
Grade 2	17	50.0	5	23.8	10	35.7	2	28.6	3	11.5	
Grade 3	11	32.4	16	76.2	18	64.3	5	71.4	22	84.6	
Age (yrs), mean (SD)(95% CI)	57.1 (13.9) (52.2 - 61.9)		57.8 (16.6) (50.2 - 65.3)		57.3 (13.9) (51.9 - 62.7)		54.1 (15.0) (40.2 - 67.9)		53.9 (12.7) (48.7 - 58.9)		0.847**

* Fisher's exact test; ** ANOVA

The association between molecular subtype and tumor grade was statistically and practically significant (p<0.001; Cramér´s V=0.337). Based on one sample Chi-square analysis, both luminal (p<0.001) and non-luminal molecular subtypes (p<0.001) were significantly associated with tumor grade 2 and 3. Table 4 depicts the mean Ki-67 values by molecular subtypes, stratified by menopausal status. There was no statistically significant difference in the mean Ki-67 between the pre- and post-menopausal groups per molecular subtypes.

**Table 4:** mean KI-67 per molecular subtypes stratified by menopausal status

Molecular subtypes	Pre-menopausal (N=43)		Pre-menopausal Ki-67	Post-menopausal (N=73)		Post-menopausal Ki-67	
	**n**	**%**	**Mean (SD) (95% CI)**	**n**	**%**	**Mean (SD) (95% CI)**	P-value*
Luminal A	12	27.9	16.3 (6.1) (12.4 - 20.1)	22	30.1	12.2 (6.3) (9.4 - 15.0)	0.081
Luminal B, HER2+	7	16.3	40.0 (14.2) (26.9 - 53.0)	14	19.2	30.0 (12.7) (22.7 - 37.3)	0.118
Luminal B, HER2-	11	25.6	45.1 (15.1) (34.9 - 55.2)	17	23.3	49.5 (21.3) (38.9 - 60.5)	0.525
HER2+ enriched	3	7.0	21.7 (14.4) (14.2 - 57.5)	4	5.5	61.3 (38.4) (18.0 - 122.3)	0.156
Triple-negative	10	23.3	53.4 (27.4) (33.8 - 73.0)	16	21.9	38.4 (27.9) (23.6 - 53.3)	0.193

* student's t-test

## Discussion

In this study, we found that 71.5% of patients had luminal molecular subtype breast cancer (luminal A, 29.3% and luminal B, 42.2%), compared to 28.5% of non-luminal breast cancer (triple-negative, 22.5% and HER2-enriched, 6.0%). Furthermore, the results revealed that 71.6% of patients were ER+, 64.7% were PR+ and 75.9% were HER2 negative. The study on global burden and trends in pre- and post-menopausal breast cancer found a growth in the incidence of ER receptor-positive tumors and a shift in the molecular subtypes prevalence towards luminal subtypes [18]. Similar to Ihemelandu *et al*. [19], we did not find any difference in molecular subtypes by menopausal status.

Across Africa, the most recent studies have shown that two-thirds of primary invasive breast cancers are luminal and that the percentage of triple-negative ranges from 15% to 30% [20-23]. Luminal molecular subtypes are generally associated with a more favorable prognosis and typically show less frequent and less extensive lymph nodal involvement than non-luminal subtypes [24]. Triple-negative breast cancer is mainly characterized by an unfavorable prognosis, with a higher risk of disease recurrence [24]. Invasive lobular carcinoma (ILC) is the second most common histological type of invasive breast carcinoma after invasive ductal carcinoma. It accounts for 10-15% of primary invasive breast cancer [25]. In our study, only 3.4% were lobular carcinoma. ILC is usually receptors positive and predominantly luminal A subtype with low proliferation index, good prognosis, good clinical response to hormonotherapy and poor response to chemotherapy [26,27]. All invasive lobular carcinomas (4/116) in our study population were ER receptor-positive equally subdivided in luminal A and luminal B. This is a small number from which major conclusions should not be drawn; however, it confirms the findings of other authors that ILC are hormone-sensitive tumors [28].

Although triple-negative breast cancer is known for its aggressive characteristics, a subgroup of triple-negative (basal-like) responds very well to neoadjuvant therapy with a complete pathological response [29]. It is now also known that the initial six molecular triple-negative subgroups of Lehmann were refined in four heterogeneous subgroups with slightly different clinical, biological and prognostic features (basal-like 1, basal-like 2, mesenchymal and luminal-androgen receptor) [30]. We found 22.4% of triple-negative breast cancer in our study. However, we could not sub-categorize in subgroups because of the retrospective nature of the data and subsequent lack of gene expression profiling.

With the cut-offs as per the 2015 St Gallen classification [15], more than half of patients presented with a high proliferation index. In general, breast cancers expressing high levels of Ki-67 correlate with worse outcomes [31,32] and shorter disease-free periods [33]. Patients with a high Ki-67 index should have chemotherapy in their treatment regimen, as tumors with a higher Ki-67 index frequently respond better to it. Nevertheless, breast cancer with a higher Ki-index is associated with poor outcomes [34]. In our study, 55.2% had a higher Ki-67 index. The stage of breast cancer is one of the key elements determining the management and the outcome of breast cancer. According to Zhang *et al*. [35], luminal A breast cancer is associated with early-stage breast cancer. In our study, most patients presented at later stages (stages II to IV) with significantly more patients in stage III. Late stages presentation was also found by McCormack *et al*. in over 1200 consecutive public hospital patients in Soweto, South Africa (54% stage III and IV) [36]. We did not, however, find any association between breast cancer stage and molecular subtypes.

Tumor size is not a key element in subtype classification and has no prognostic attribute in this classification. However, it is a key component in the Nottingham prognostic index score [37]. Regarding the association of tumor size and molecular subtypes, our finding is similar to Errahhali *et al*. [38], in that there was no association between tumor size and molecular subtypes. Rahmawati *et al*. [39] in their study among Indonesian women also did not find any association between molecular subtypes and tumor size. Similar to what has been found in other studies [40,41], we found a statistical and moderate clinically significant association between tumor grade and molecular subtype. High-grade tumors were associated with triple-negative breast cancer. This loss of estrogen receptor in advanced diseases was also found in a systematic review of molecular subtypes in indigenous populations in Africa [42]. Pareja *et al*. [43] found that most triple-negative cases were very invasive with a high-grade tumor. Pareja *et al*. also reported a small subset with low grade and indolent clinical progression. Higher grade tumors are more likely to be receptor-negative.

The presence of axillary lymph nodes signs the progress of the disease beyond the primary tumor. Because of late presentation, most of the breast cancer in developing countries presents with axillary lymph node involvement. Basro and Apffelstaedt [44], found that 64.8% of patients in South Africa presented with positive lymph nodes at diagnosis. We found in our study that 67% of patients had axillary lymph node metastases on histology results. Axillary lymph nodes metastasis involvement was observed across all different molecular subgroups as follows: luminal A 67.6% (25/37), luminal B HER2+ 80% (16/20), luminal B HER2- 55.6% (15/27), HER2+ enriched 85.7% (6/7) and triple-negative, 65.4% (17/26). According to Si *et al*. [45], tumor size has a stronger correlation with axillary lymph node status than molecular subtypes. The axillary lymph node involvement across all subtypes in our study reflected the disease progression and was not influenced by the molecular subtypes.

There are several potential limitations to this study. Firstly, missing data of other biomarkers such as cytokeratin 5/6, cytokeratin 14, EGFR and P53, on the histology report did not allow for the subdivision of triple-negative cases into sub-groups. Secondly, the inexistence of gene expression profiling in our setting did not allow us to determine the prevalence of molecular subtypes with more accuracy using GEP. Thirdly, because of the lack of data on the age of onset of menopause, we used the age of 50 years as a cut-off in the study as a proxy. Despite these limitations, this study is the first on molecular subtypes and receptor status in the Potchefstroom Hospital catchment area.

## Conclusion

The understanding and identification of molecular subtypes of breast cancer are important in the management thereof since they predict the prognosis and clinical outcome. Our study showed that most of the breast cancer patients in our setting were receptor-positive and approximately a quarter of patients were triple-negative. Furthermore, the study showed that luminal types A and B were the preponderant subtypes. Molecular subtypes were associated with tumor grade but independent of age and menopausal status. The results of this study will be used to optimize treatment protocols and personalized management strategies for breast cancer patients in the Potchefstroom Hospital catchment area.

### What is known about this topic

- Breast cancer is a heterogeneous disease with different molecular subtypes;
- Molecular subtypes have differing disease progression with contrasting response to different treatment modalities;
- Receptor-positive breast cancers have a good clinical response to hormonal therapy.

### What this study adds

- This is the first study on receptor status and molecular subtypes in Potchefstroom Hospital and one of the few published in South Africa on this topic;
- This study shows that more than three-quarters of breast cancer in our setting is receptor-positive as opposed to some earlier African studies which reported the opposite.
